# Gene polymorphisms of *METTL5* and *METTL16* are related to epithelial ovarian cancer risk in South China: A three-center case-control study

**DOI:** 10.7150/jca.90379

**Published:** 2024-02-04

**Authors:** Siyi Zhang, Shanshan Liu, Wenchu Chen, Yaping Yan, Mansi Cai, Xiaoping Liu, Ailing Luo, Wenjuan Li, Lisha Yi, Yingyi Xu

**Affiliations:** 1 Department of Anesthesiology, Guangzhou Women and Children's Medical Center, Guangzhou Medical University, Guangdong Provincial Clinical Research Center for Child Health, Guangzhou, 510623, China.; 2 Department of Hematology and Oncology, Guangzhou Women and Children's Medical Center, Guangzhou Medical University, Guangdong Provincial Clinical Research Center for Child Health, Guangzhou, 510623, China.; 3 Medical Research Center, Shunde Hospital, Southern Medical University, Foshan, Guangdong, 528000, China.; 4 Department of gynaecology, Guangzhou Women and Children's Medical Center, Guangzhou Medical University, Guangdong Provincial Clinical Research Center for Child Health, Guangzhou, 510623, China.

**Keywords:** epithelial ovarian cancer, polymorphism, methyltransferase-like gene, susceptibility

## Abstract

**Background**: The potential relation of methyltransferase-like gene polymorphisms and epithelial ovarian cancer (EOC) remains unclear.

**Methods:** Five SNPs (*METTL5* rs3769767 A>G, *METTL16* rs1056321 T>C, *METTL5* rs10190853 G>A, *METTL5* rs3769768 G>A and *METTL16* rs11869256 A>G) of methyltransferase-like genes was selected trough NCBI dbSNP database. Two hundred and eighty-eight cases and 361 controls were enrolled from three hospitals in South China to conduct the case-control study. Genomic DNA was abstracted from peripheral blood and genotyped through a TapMan assay. Stratified analysis was conducted to explore the association of rs10190853, rs3769768, rs11869256 genotype and EOC susceptibility. The combination analysis was adopted to evaluate the relation between inferred haplotypes of the *METTL5, METTL16* genes and EOC risk. Multifactor dimensionality reduction (MDR) analysis was performed to verify the interaction of SNPs.

**Results:** Among the five analyzed SNPs, *METTL5* rs3769768 AA exhibited a significant association with increased EOC risk, while *METTL5* rs10190853 GA, *METTL16* rs11869256 GA was certified to decrease the susceptibility of EOC. The stratified analysis further revealed the harmful effect of *METTL5* rs3769768 AA in EOC patients. On the contrary, *METTL16* rs11869256 AG/GG and *METTL5* rs10190853 AA showed the reduced risk of EOC in patients of specific subgroups. Combination analysis identified that haplotypes AAA highly connected with reduced risk of EOC. MDR analysis revealed that these SNPs existed no specific interactions.

**Conclusion**: *METTL5* rs3769768 was related to increased risk of EOC. *METTL5* rs10190853 and *METTL16* rs11869256 decreased the susceptibility in EOC. *METTL5* and *METTL16* could be potential target of molecular therapy and prognosis markers.

## Introduction

Epithelial ovarian cancer (EOC) remains the most common cause of death amongst all type of gynaecological malignancies, accounts for 90% of all ovarian cancers. It occurs about 57200 new cases and 27200 cases of death in China yearly, which comprise 8.47% of all cancers and 4.04% of all cancer deaths, respectively^1^. The 5-years relative survival of EOC is 50.8%^2^. The diagnostic evaluation of EOC involves pelvic ultrasound, computed tomography (CT) scans of the thorax, abdomen, and pelvis, and pathological examination. Additionally, serum levels of CA-125, CA 19-9, and CEA are crucial for the diagnosis^3^. EOC is classified as high-grade serous (HGSOC), low-grade serous(LGSOC), mucinous, clear cell, endometroid (EC), and undifferentiated according to pathological features^4^, ^5^. Based on the International Federation of Gynecology and Obstetrics (FIGO 2018) system, EOC staging range from I to IV. Optimal surgery and platinum-based chemotherapy contributes to cure of early stage EOC with 92.4% 5-year survival rate, but can't improve the prognosis of advanced stage^6^.

N6-methyladenosine (m6A) is the most abundant internal modification of RNA in eukaryotic cells, which has been proved to have impact on carcinogenesis. Methyltransferases METTL3, METTL14, METTL5, METTL16 and Wilms' tumor 1-associated protein (WTAP), demethylases fat mass and obesity-associated protein (FTO) and α-ketoglutarate-dependent dioxygenase homolog 5 (ALKBH5), and readers YT521-B homology (YTH) domain family (including YTHDF1, YTHDF2, YTHDF3, YTHDC1 and YTHDC2) play essential roles during the modification. Targeting on m6A regulators promotes the development of anticancer drugs^7^. The m6A methyltransferases participant in the tumor growth, metastasis, and chemotherapy resistance of ovarian cancer^8^. METTL3 can promote the tumor growth of EOC^9^. Studies have shown that in EOC, m6A methylation regulators modify the tumor progression, such as high expression of WTAP relating to the poor prognosis of EOC^8^. Chang *et al.*^10^ assessed the METTL16 expression in EOC and found that low expression of METTL16 was connected with shorter overall survival, which suggested that METTL16 might be beneficial to prognostic evaluation of EOC, but the specific mechanism remains unclear. However, the expression and function of ribosomal RNA m6A methyltransferase METTL5 in EOC has not been reported.

Genome-wide association studies (GWAS) have identified several loci and single nucleotide polymorphisms (SNPs) that are linked to the susceptibility of EOC^11^, ^12^. *METTL3* polymorphisms reduced the susceptibility of Wilms tumor^13^. It was reported that *METTL14* polymorphisms may relate to risk of hepatoblastoma^14^. *WTAP* polymorphisms has weak impact on the susceptibility of neuroblastoma^15^. Zhen *et al.*^16^ found that *METTL16* polymorphisms reduced the risk of sudden cardiac death. As far as we know, no study conducted to verify the potential association of *METTL16* polymorphisms and cancers. Besides, there is no research reveal the relationship between *METTL5* polymorphisms and diseases. A plenty of studies discover that heritable variations in genes have remarkable effect on the progression of EOC^17^. Nevertheless, how SNPs in m6A methyltransferases METTL5 and METTL16 function on the risk of EOC remains unrevealed.

In present study, we aimed to reveal the association between methyltransferase *METTL5* and *METTL16* gene polymorphisms and EOC susceptibility by performing a three-center case-control study in South China, which included five SNPs.

## Materials and Methods

### Study subjects

In our study, 288 EOC patients and 361 controls were enrolled from Guangzhou Women and Children's Center, The First Affiliated Hospital of Jinan University, and Shunde Hospital of Southern Medical University. Age-matched control subjects, who were free of EOC or other gynaecological malignancies, were collected from volunteers visiting the same hospital. All participants signed consent to use their sample for research purpose. The study was approved by the Ethics Committee of Guangzhou Women and Children's Medical Center (No.117A01). The demographic characteristics of all participants were presented in Table S1.

### SNP selection and genotyping

We performed the selection of potentially functional SNPs through the NCBI dbSNP database (http://www.ncbi.nlm.nih.gov/projects/SNP) and the SNP info (https://snpinfo.niehs.nih.gov/snpinfo/snpfunc.html) according to the following criteria: located in the 5' untranslated region, 3' untranslated region, 5' flanking region, and exon of the gene, low linkage disequilibrium (R^2^ < 0.8). Five SNPs (rs3769767 A>G, rs1056321 T>C, rs10190853 G>A, rs3769768 G>A, rs11869256 A>G) were chosen. DNA was extracted by the TIANamp DNA Kit (Tiangen, Beijing, China) according to the standard instructions of the kit. In order to exclude genotyping errors, genotyping results were confirmed by randomly assaying 10% of the original specimens for replication.

### SNP‑SNP interaction analysis

Epistasis was evaluated and characterized between the SNPs by the multifactor dimensionality reduction (MDR) method using the MDR software, v3.0.2 (Computational Genetics Laboratory, University of Pennsylvania, USA; available for free at https://www.epistasis.org) as previously described^18^. Briefly, the genotype of each SNP was characterized by a predefined number and analyzed in conjunction with data indicating the presence or absence of EOC. Cross-validation consistency (CVC) and the test accuracy were used to identify the best interaction models. Values with a P <0.05 were considered statistically significant.

### Statistical analysis

The χ^2^ test was performed to assess if the selected *METTL5* and *METTL16* SNPs deviated from Hardy-Weinberg equilibrium among controls. The two-sided χ^2^ test was used to compare demographic variables and genotype frequencies of the cases and controls. Unconditional logistic regression analyses were conducted to compute ORs and their corresponding 95% CIs with or without adjustment for age. The SAS statistical package (version 9.1; SAS Institute, Cary, NC) was adopted to perform all statistical analyses. A two-sided P values was used for all the statistical analysis and a P value < 0.05 was considered as statistical significance.

## Results

### Association of m6A methyltransferases polymorphisms and EOC risk

Our study successfully genotyped five SNPs, which were rs3769767 A/G, rs1056321 T/C, rs10190853 G/A, rs3769768 G/A, and rs11869256 A/G. The single-locus analysis was conducted to identify the association between five SNPs and EOC risk, as shown in Table 1. The *METTL5* rs10190853 (GA versus GG: adjusted OR=0.607,95% CI=0.432-0.853, P=0.0041) and *METTL16* rs11869256 (GA versus AA: adjusted OR=0.565,95% CI=0.401-0.796, P=0.0011) variant alleles showed reduced susceptibility to EOC. On the contrary, the *METTL5* rs3769768 (AA versus GG: adjusted OR=2.841,95% CI=1.026-7.867, P=0.0444) variant alleles resulted in enhanced risk of EOC. The rest two SNPs of *METTL5* gene (rs3769767 A>G, rs1056321 T>C) were not detected relationship with EOC risk.

### Stratification analysis of identified SNPs

The further stratification analysis was applied to screening the connection of three significant risk-associated SNPs (rs10190853 G/A, rs3769768 G/A, rs11869256 A/G) and clinical parameters of EOC (Table 2), including age, metastasis, clinical stage, pathological grade, tumor number, tumor size, pregnancy history, and expression levels of ER, PR, PAX8, wild-type p53, mutant p53, WT1, P16 and ki67. For the *METTL5* rs10190853, a significant decreased risk of EOC was revealed among patients with negative/mild positive WT1 expression (adjusted OR=2.225, 95%CI=1.009-4.909, P=0.0476). Besides, the *METTL16* rs11869256 decreased EOC risk among patients older than 53 years old (adjusted OR=0.440, 95% CI=0.252-0.769, P=0.004), metastasis (adjusted OR=0.531, 95% CI=0.329-0.856, P=0.0094), no metastasis (adjusted OR=0.612, 95% CI=0.408-0.917, P=0.0174), clinical stage II (adjusted OR=0.416, 95% CI=0.221-0.78, P=0.0063), clinical stage III (adjusted OR=0.552, 95% CI=0.337-0.903, P=0.0180), high pathological grade (adjusted OR=0.596, 95% CI=0.400-0.887, P=0.0108), multiple tumor number (adjusted OR=0.604, 95% CI=0.384-0.950, P=0.0291), tumor size larger than 3 cm (adjusted OR=0.524, 95% CI=0.303-0.907, P=0.0209), tumor size ≤ 3 cm (adjusted OR=0.604, 95% CI=0.410-0.889, P=0.0105), pregnant times more than 3 times (adjusted OR =0.463, 95% CI=0.298-0.719, P=0.0006), post-menopause (adjusted OR=0.495, 95% CI=0.333-0.735, P=0.0005), high wild p53 expression (adjusted OR=0.531, 95% CI=0.366-0.770, P=0.0008), mutant p53 expression (adjusted OR=0.608, 95% CI=0.393-0.942, P=0.0258), without mutant p53 expression (adjusted OR=0.56, 95%CI=0.37-0.547, P=0.0061), negative/mild positive WT1 expression (adjusted OR=0.405, 95% CI=0.200-0.819, P=0.0119), high WT1 expression (adjusted OR=0.555, 95% CI=0.340-0.908, P=0.017), negative/mild positive P16 expression (adjusted OR=0.479, 95% CI=0.234-0.981, P=0.0443), negative/mild positive ki67 expression (adjusted OR=0.432, 95% CI =0.219-0.854, P=0.0157). In contrary, the *METTL5* rs3769768 enhanced the risk of EOC in patients with metastasis (adjusted OR=4.965, 95% CI=1.598-15.421, P=0.0056), clinical stage 2 (adjusted OR=5.725, 95% CI=1.508-24.731, P=0.0103), low pathological grade(adjusted OR=5.803, 95%CI=1.779-18.923, P=0.0036), tumor size > 3 cm (adjusted OR=4.453, 95%CI=1.298-15.271, P=0.0175), post-menopause (adjusted OR=4.296, 95% CI=1.464-12.608, P=0.008), high wild p53 expression (adjusted OR=3.177, 95% CI=1.122-8.997, P=0.0296), no mutant p53 expression (adjusted OR=3.699, 95% CI=1.243-11.007, P=0.0187).

### Association between inferred haplotypes of the m6A methyltransferases genes polymorphisms and EOC risk

We further evaluated if haplotypes of the *METTL5* and *METTL16* genes SNPs rs10190853 G>A, rs3769768 G>A, rs3769768 G>A, were linked to EOC risk. As shown in Table 3, the haplotypes GAG was characterized as reference group. The haplotypes AAA (adjusted OR=0.662, 95% CI=0.466-0.941, P=0.021) contributed to reduce the risk of EOC.

### Expression quantitative trait loci (eQTL) analyses

**Figure 1:**
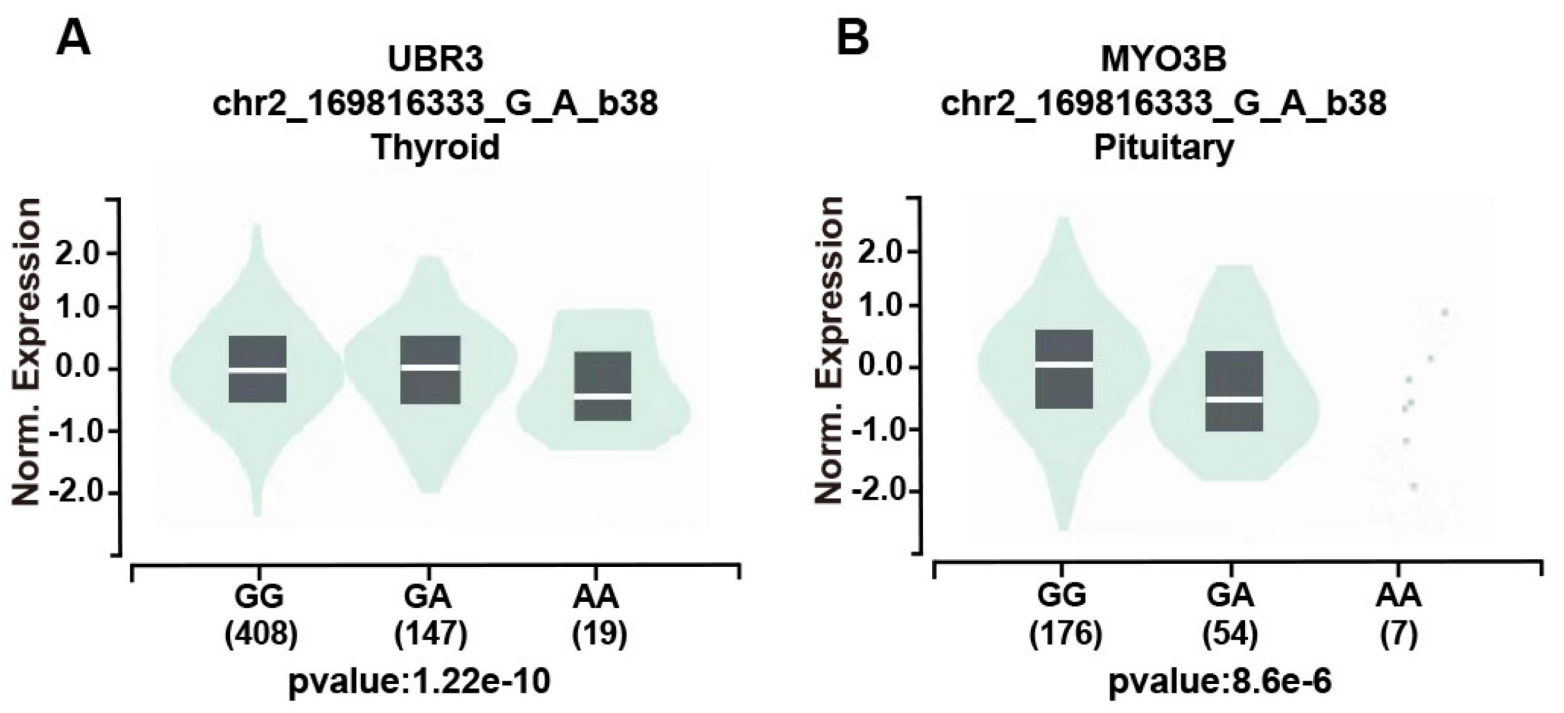
** The effect of the rs10190853 gene and its adjacent gene UBR3 and MYO3B polymorphisms according to the GTEx public database.** Different genotypes influence the expression of the rs10190853 gene and *UBR3* and *MYO3B* in distinct organs and tissues. (A) The expression of *UBR3* with different rs10190853 genotypes was shown in the thyroid. (B) The expression of *MYO3B* with different rs10190853 genotypes was shown in the pituitary.

We further assessed the potential functional relevance of five SNPs using released data from GTEx (https://www.gtexportal.org/home/index.html) while rs3769767 A>G, rs1056321 T>C, rs10190853 G>A, rs3769768 G>A, rs11869256 A>G were not significantly related to expression of *METTL5*, *METTL16*. However, the rs10190853 AA genotype reduced the expression of *MYO3B* in pituitary, which is a neighboring gene of *METTL5* (Figure 1A), and the expression of another adjacent gene *UBR3* in thyroid (Figure 1B).

### SNP‑SNP interaction analysis

**Figure 2:**
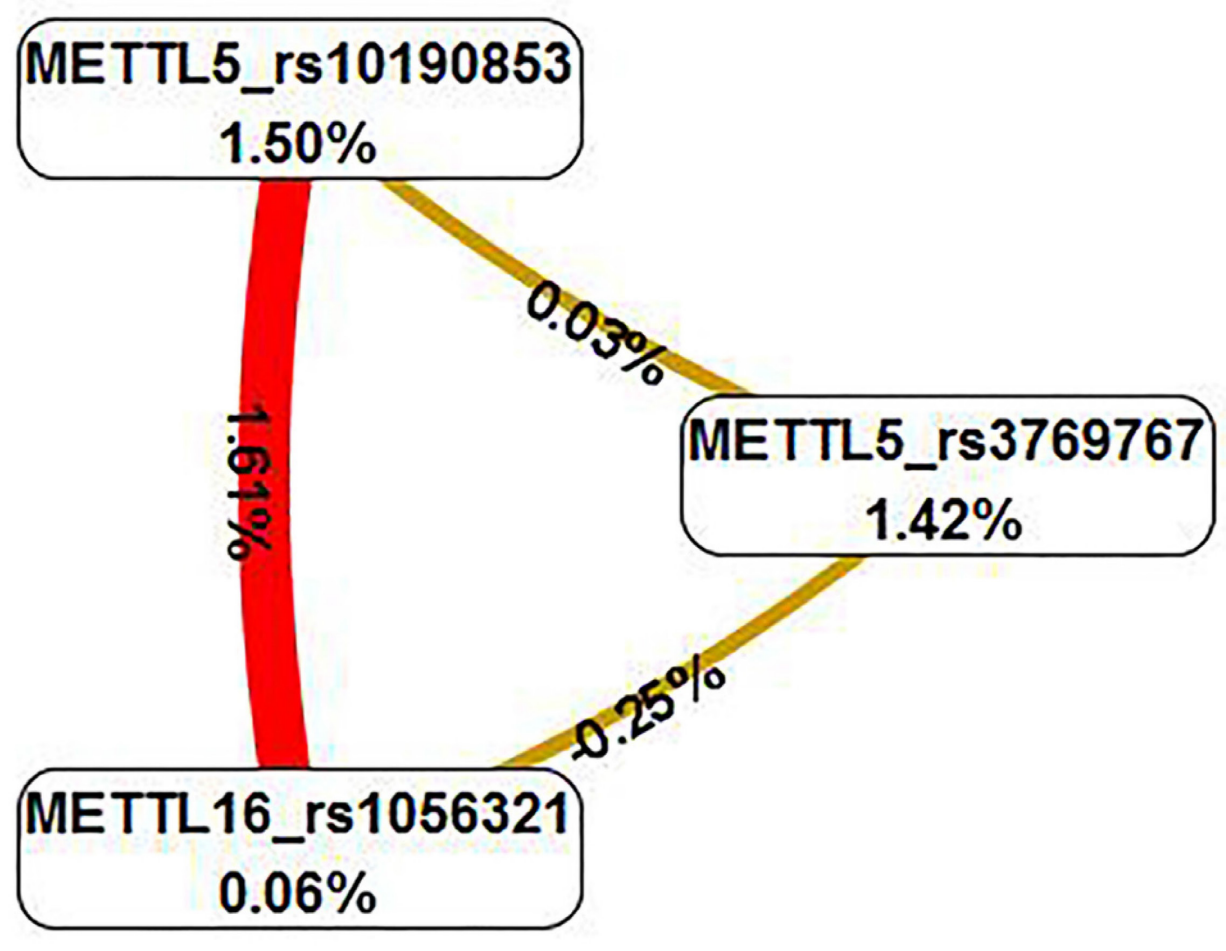
** Interaction map for EOC risk.** The interaction model describes the percentage of the entropy (information gain) that is explained by each factor or 2-way interaction. Positive entropy (plotted in red) indicates interaction, which can be interpreted as a synergistic or nonadditive relationship; while negative entropy (plotted in yellow-green or green) indicates independence or additivity (redundancy).

The multifactor dimensionality reduction analysis was conducted to verify the interaction of SNPs, which presented the interaction model of rs3769767 of the *METTL5* gene, rs1056321 of the *METTL16* gene, rs10190853 of the *METTL5* gene, and a CVC values 10/10, test accuracy=0.6191, OR=2.505, 95% CI=0.8889-7.0587, P=0.0793 (Table 4). The interaction map showed the following interaction: *METTL5*_rs10190853 × *METTL16*_rs1056321, with high values of positive entropy or synergism (1.61%, shown in red); low entropy values mean redundancy or even independence (Figure 2).

## Discussion

In our case-control study, 288 EOC patients and 361controls in South China were enrolled to reveal the latent connection between *METTL5 and METTL16* gene polymorphisms and EOC risk. Three polymorphisms were demonstrated to link with the susceptibility of EOC, namely rs10190853 G>A, rs3769768 G>A, and rs11869256 A>G. Among these polymorphisms, rs10190853 G>A and rs11869256 A>G decreased the risk of EOC, while rs3769768 G>A enhanced the susceptibility of EOC. To our knowledge, it was the first time to explore the underlying relation of m6A methyltransferase gene *METTL5* and* METTL16* polymorphisms and susceptibility of EOC.

It was widely reported that m6A modification plays a significance role in the progression and prognosis of EOC. During the process of methylation, methyltransferases act as writers to regulate m6A methylation of mRNA^19^. Ribosomal RNA m6A* METTL5* might influence the carcinogenesis of renal cancer through modifying the immune microenvironment^20^. Sun *et al.*^21^ revealed that high expression of *METTL5* was associated with poor prognosis of lung adenocarcinoma. As yet, no researches have revealed the association between *METTL5* and EOC.

METTL16 promoted expression of branched-chain amino acid (BCAA), transaminase 1 (BCAT1) and BCAT2 through m6A-dependent manner and reprogramed BCAA metabolism in acute myeloid leukemia.^22^ In 2022, Ye *et al.*^23^ found that METTL16 inhibited ferroptosis to enhance the expression of GPX4, resulting in breast cancer progression. It was reported that METTL16 played a suppressor role in pancreatic adenocarcinoma by METTL16-p21 signaling axis.^23^, ^24^ High expression of METTL16 predicted a poor outcome of soft-tissue sarcomas.^25^ To date, only a limited number of studies have been conducted to elucidate the association between METTL16 and EOC, which have revealed compelling evidence suggesting that METTL16 may function as a tumor suppressor in the progression of EOC^10^.

Wang *et al*.^26^ reported that METTL5 mutant could lead to craniofacial neural development defects in mouse embryonic stem cell. Bi-allelic variants in METTL5 cause intellectual disability and microcephaly in human^27^. Copy number variation of METTL16 is abundant in bladder cancer patients with mutant TP53^28^. Inactivating frameshift mutation of METTL16 accelerate the tumorigenesis in colorectal cancer^29^. Nevertheless, no research has been exerted to clarify the association of *METTL5*, *METTL16* polymorphisms and EOC. We predicted the location and function of these selected SNPs through online software SNP info. *METTL5* rs10190853 G>A and rs3769768 G>A polymorphism were located in chromosome 2, which were predicted to be exon splicing enhancer or silencer, transcription factor binding sites, respectively. *METTL16* rs11869256 A>G polymorphism was located in chromosome 17 and was predicted to be transcription factor binding sites. Our study proved that *METTL5* rs10190853 GA phenotypes and *METTL16* rs11869256 GA phenotypes decreased the risk of EOC, while *METTL5* rs3769768 AA phenotypes was linked with enhancive susceptibility of EOC, which were different from the previous researches. It was likely to due to the *METTL5* and *METTL16* genetic variations function diversely on multiple cancers, and influence the progression of EOC through transcription modification.

P53 is a tumor suppressor involving in metabolism regulation and diverse aspects of differentiation and progression^30^. LGSOC usually express wild *p53*, while HGSOC is characterized by expression of mutant p53^31^. Abnormal p53 expression was related to poor prognosis of clear cell carcinoma^32^. It was reported that *METTL5* could prevent mutant p53 from undergoing ubiquitination-dependent degradation through HSF4b/HSP90B1 pathway to facilitate nasopharyngeal carcinoma tumor progression^33^. There exist no researches conducted to verify the association between *METTL5, METTL16* and expression of *p53* in EOC patients. Wilms' tumor gene WT1 is a transcriptional regulator targeting genes^34^. WT1 acts as tumor-suppressor as well as oncogene in diverse cancers^35^. It was reported that WT1 could modify E-cadherin and ERK1/2 pathway to enhance the progression of ovarian cancer^36^. As far as we know, no researches have been conducted to reveal the association of* METTL5*, *METTL16* and *WT1* expression in EOC. Ki67 is a well-known proliferation marker and could be the target of cancer therapy^37^. It was reported that high expression of ki67 related to poor prognosis of EOC^38^. Our study found that the rs3769768 AA enhanced the EOC risk in patients with strong positive wild p53 expression and no mutant p53 expression. On the contrary, the rs10190853 AA played a decreased role in susceptibility of EOC in patients with negative/mild WT1 expression, which indicated that high expression of WT1 might be a marker for a poor prognosis of EOC. In addition, the rs10190853 AA allele reduced the expression of MYO3B in pituitary in eQTL analysis. It was reported that the polymorphisms of UBR3 and MYO3B were related to saccular intracranial aneurysm in Portugal^39^. UBR3 plays a stimulative role in intervertebral disc degeneration trough increase inflammation^40^. To date, there is a dearth of literature exploring the association between UBR3 and tumor diseases. MYO3B was reported that can facilitate the treatment of trastuzumab in HER2+ breast cancer^41^.Up to now, no significant association has been identified between the polymorphism of the METTL5 gene and its neighboring genes UBR3 and MYO3B. We hypothesized that the decreased expression of MYO3B caused by the AA allele in *METTL5* rs10190853 in the pituitary may relate to the hormone secretion of EOC. In our study, the rs11869256 AG/GG decreased the risk of EOC in patients with high expression of wild p53 and negative/mild expression of ki67. The MDR analysis presented no specific interaction of *METTL5* and *METTL16* polymorphism, so that they may affect the EOC independently. As far as we know, it was the first time to discover that *METTL5*, *METTL16* polymorphisms were related to risk of EOC patients with expression of wild *p53*, *WT1 and ki67*, which might provide a new target of molecular therapy in EOC.

Following limitations could not be neglected in the present study. First of all, sample size was not adequate enough and larger sample size was required for further analysis. Secondly, the exact molecular mechanism of methyltransferase-like gene influencing the susceptibility of EOC was not explored. Finally, the association between methyltransferase-like gene polymorphisms and prognosis of EOC was not evaluated.

## Conclusions

Our study proved that *METTL5* rs10190853 GA phenotypes and *METTL16* rs11869256 GA phenotypes decreased the risk of EOC, while *METTL5* rs3769768 AA phenotypes was associated with increased susceptibility of EOC, indicating that *METTL5*, *METTL16* might be a potential target of molecular therapy and prognosis marker.

## Supplementary Material

Supplementary table.

## Figures and Tables

**Table 1:** Logistic regression analysis of associations between *METTL5, METTL16* polymorphisms and EOC susceptibility.

Genotype	Cases	Controls	P^a^		Crude OR			P	Adjusted OR			P^b^
	(N=288)	(N=361)			(95% CI)				(95% CI) b			
rs3769767A>G (HWE=0.001)												
AA	163 (69.07)	182 (56.88)		1.00						1.00		
AG	62 (26.27)	132 (41.25)		0.553 (0.389-0.786)				0.0010		0.548 (0.384-0.782)	0.0009	
GG	11 (4.66)	6 (1.88)		2.157 (0.785-5.930)				0.1361		2.483 (0.895-6.884)	0.0805	
Additive			0.0004	0.729 (0.533-0.996)				0.0472		0.733 (0.534-1.007)	0.0555	
Dominant	73 (30.93)	138 (43.13)	0.0034	0.591 (0.415-0.841)				0.0035		0.584 (0.409-0.836)	0.0033	
Recessive	225 (95.34)	314 (98.13)	0.0593	2.560 (0.933-3.024)				0.0680		3.005 (1.083-8.339)	0.0346	
rs1056321T>C (HWE=0.082)												
TT	124 (47.88)	149 (44.88)		1.00						1.00		
TC	103 (39.77)	136 (40.96)		1.030 (0.740-1.433)				0.8626		1.072 (0.768-1.498)	0.6826	
CC	32 (12.36)	47 (14.16)		0.926 (0.564-1.519)				0.7597		0.919 (0.557-1.515)	0.7391	
Additive			0.7091	0.906 (0.717-1.144)				0.4075		0.915 (0.723-1.158)	0.4591	
Dominant	135 (52.12)	183 (55.12)	0.4684	0.886 (0.640-1.228)				0.4685		0.914 (0.658-1.271)	0.5943	
Recessive	227 (87.64)	285 (85.84)	0.5231	0.855 (0.528-1.384)				0.5234		0.832 (0.511-1.354)	0.4592	
rs10190853 G>A (HWE=0.322)												
GG	103 (44.98)	152 (41.87)		1.00						1.00		
GA	86 (37.55)	159 (43.80)		0.601 (0.429-0.842)				0.0031		0.607 (0.432-0.853)	0.0041	
AA	40 (17.47)	52 (14.33)		0.855 (0.538-1.359)				0.5069		0.825 (0.516-1.318)	0.4214	
Additive			0.2822	1.001 (0.794-1.261)				0.9951		0.985 (0.780-1.245)	0.9001	
Dominant	126 (55.02)	211 (58.13)	0.4575	0.881 (0.631-1.230)				0.4576		0.875 (0.624-1.226)	0.4364	
Recessive	189 (82.53)	311 (85.67)	0.3041	1.266 (0.807-1.985)				0.3048		1.209 (0.766-1.907)	0.4155	
rs3769768 G>A (HWE=0.709)												
GG	182 (72.51)	244 (76.25)		1.00						1.00		
GA	58 (23.11)	70 (21.88)		1.192 (0.808-1.757)				0.3759		1.213 (0.820-1.796)	0.3341	
AA	11 (4.38)	6 (1.88)		2.637 (0.961-7.237)				0.0598		2.841 (1.026-7.867)	0.0444	
Additive			0.1892	1.267 (0.918-1.747)				0.1495		1.300 (0.940-1.798)	0.1132	
Dominant	69 (27.49)	76 (23.75)	0.3082	1.217 (0.834-1.777)				0.3085		1.249 (0.852-1.829)	0.2542	
Recessive	240 (95.62)	314 (98.13)	0.0802	2.399 (0.875-6.578)				0.0892		2.558 (0.926-7.068)	0.0700	
rs11869256 A>G (HWE=0.223)												
AA	109 (42.91)	103 (30.47)		1.00						1.00		
GA	91 (35.83)	177 (52.37)		0.561 (0.399-0.788)				0.0008		0.565 (0.401-0.796)	0.0011	
GG	54 (21.26)	58 (17.16)		1.016 (0.658-1.569)				0.9441		1.071 (0.690-1.662)	0.7599	
Additive			0.0003	0.851 (0.678-1.068)				0.1637		0.865 (0.688-1.089)	0.2167	
Dominant	145 (57.09)	235 (69.53)	0.0018	0.583 (0.415-0.819)				0.0019		0.588 (0.417-0.829)	0.0024	
Recessive	200 (78.74)	280 (82.84)	0.2074	1.303 (0.863-1.969)				0.2081		1.364 (0.898-2.072)	0.1452	
												

Abbreviations: EOC, epithelial ovarian cancer; HWE, Hardy-Weinberg equilibrium; OR, odds ratios; CI, confidence interval; FIGO, International Federation of Gynaecology and Obstetrics; NA = not applicable. ^a^ χ2 test for genotype distributions between EOC cases and cancer‐free controls. ^b^ Adjusted for age.

**Table 2:** Stratification analysis of *METTL5, METTL16* polymorphisms with EOC susceptibility.

Variables	rs10190853 G>A		Adjusted OR ^a^	*P* ^a^	rs3769768 G>A		Adjusted OR ^a^	*P* ^a^	rs11869256 A>G		Adjusted OR ^a^	*P* ^a^
	(cases/controls)		(95% CI)		(cases/controls)		(95% CI)		(cases/controls)		(95% CI)	
	GG/GA	AA			GG/GA	AA			AA	AG/GG		
Age, years												
>53	86 (311)	21 (52)	1.172 (0.595-2.309)	0.6459	110 (314)	5 (6)	>999.999 (<0.001, >999.999)	0.9792	54 (103)	61 (235)	0.440 (0.252-0.769)	0.0040
≤53	103 (311)	19 (52)	1.239 (0.671-2.291)	0.4935	130 (314)	6 (6)	1.585 (0.500-5.018)	0.4338	55 (103)	84 (235)	0.706 (0.458-1.094)	0.1194
Metastasis												
Yes	72 (311)	15 (52)	1.213 (0.642-2.290)	0.5519	84 (314)	7 (6)	4.965 (1.598-15.421)	0.0056	41 (103)	50 (235)	0.531 (0.329-0.856)	0.0094
No	103 (311)	22 (52)	1.213 (0.699-2.108)	0.4923	138 (314)	3 (6)	1.309 (0.320-5.359)	0.7085	61 (103)	85 (235)	0.612 (0.408-0.917)	0.0174
Clinical stage												
I	32 (311)	11 (52)	0.903 (0.893-4.052)	0.0954	49 (314)	3 (6)	4.013 (0.930-17.309)	0.0624	20 (103)	35 (235)	0.768 (0.419-1.409)	0.3944
II	32 (311)	8 (52)	1.454 (0.633-3.340)	0.3778	41 (314)	4 (6)	5.725 (1.508-24.731)	0.0103	23 (103)	22 (235)	0.416 (0.221-0.780)	0.0063
III	75 (311)	12 (52)	0.93 (0.474-1.851)	0.8509	85 (314)	2 (6)	1.305 (0.257-6.635)	0.7486	37 (103)	47 (235)	0.552 (0.337-0.903)	0.0180
IV	19 (311)	3 (52)	0.919 (0.262-3.224)	0.8948	22 (314)	1 (6)	2.643 (0.296-23.601)	0.3844	9 (103)	14 (235)	0.681 (0.286-1.626)	0.3873
Pathological grade												
low	49 (311)	12 (52)	1.359 (0.672-2.750)	0.3933	63 (314)	6 (6)	5.803 (1.779-18.923)	0.0036	30 (103)	45 (235)	0.651 (0.387-1.094)	0.1048
high	128 (311)	24 (52)	1.076 (0.632-1.831)	0.7883	151 (314)	5 (6)	1.852 (0.551-6.227)	0.3189	66 (103)	89 (235)	0.596 (0.400-0.887)	0.0108
Tumor number												
single	51 (311)	13 (52)	1.437 (0.720-2.868)	0.3041	76 (314)	3 (6)	2.504 (0.593-10.573)	0.2118	36 (103)	51 (235)	0.617 (0.346-3.011)	0.0553
multiple	89 (311)	12 (52)	0.794 (0.406-1.555)	0.5017	104 (314)	3 (6)	1.592 (0.389-6.515)	0.5179	44 (103)	61 (235)	0.604 (0.384-0.950)	0.0291
Tumor size												
>3 cm	50 (311)	15 (52)	1.682 (0.873-3.238)	0.1201	64 (314)	5 (6)	4.453 (1.298-15.271)	0.0175	29 (103)	35 (235)	0.524 (0.303-0.907)	0.0209
≤3 cm	120 (311)	23 (52)	1.116 (0.651-1.915)	0.6896	155 (314)	4 (6)	1.485 (0.409-5.385)	0.5478	71 (103)	99 (235)	0.604 (0.410-0.889)	0.0105
Pregnant times												
≤3	107 (311)	25 (52)	1.322 (0.777-2.249)	0.3038	140 (314)	6 (6)	2.447 (0.767-7.803)	0.1304	55 (103)	88 (235)	0.700 (0.463-1.058)	0.0903
>3	82 (311)	15 (52)	1.053 (0.561-1.976)	0.8720	100 (314)	5 (6)	2.912 (0.861-9.849)	0.0856	54 (103)	57 (235)	0.463 (0.298-0.719)	0.0006
Pausimenia												
post-menopause	136 (311)	29 (52)	1.132 (0.671-1.911)	0.6422	167 (314)	10 (6)	4.296 (1.464-12.608)	0.0080	83 (103)	96 (235)	0.495 (0.333-0.735)	0.0005
pre-menopause	53 (311)	11 (52)	1.394 (0.660-2.946)	0.3837	73 (314)	1 (6)	0.470 (0.056-3.973)	0.4884	26 (103)	49 (235)	0.871 (0.502-1.510)	0.6222
ER expression												
negative/mild positive	28 (311)	3 (52)	0.607 (0.177-2.085)	0.4280	33 (314)	1 (6)	1.883 (0.216-16.446)	0.5671	15 (103)	19 (235)	0.537 (0.261-1.105)	0.0912
strong positive	50 (311)	15 (52)	1.651 (0.857-3.182)	0.1341	65 (314)	2 (6)	1.792 (0.350-9.168)	0.4839	27 (103)	45 (235)	0.730 (0.428-1.243)	0.2460
PR expression												
negative/mild positive	27 (311)	6 (52)	1.295 (0.508-3.298)	0.5883	32 (314)	1 (6)	1.679 (0.195-14.472)	0.6374	15 (103)	20 (235)	0.580 (0.285-1.180)	0.1328
strong positive	30 (311)	6 (52)	1.204 (0.477-3.043)	1.0000	37 (314)	1 (6)	1.322 (0.153-11.406)	0.7998	10 (103)	31 (235)	1.359 (0.642-2.875)	0.4229
PAX8 expression												
negative/mild positive	22 (311)	5 (52)	1.312 (0.474-3.632)	0.6018	25 (314)	0 (6)	-		13 (103)	14 (235)	0.463 (0.210-1.024)	0.0572
strong positive	56 (311)	6 (52)	0.631 (0.258-1.541)	0.3120	63 (314)	2 (6)	1.667 (0.327-8.490)	0.5382	26 (103)	39 (235)	0.657 (0.380-1.136)	0.1328
Wild p53 expression												
negative/mild positive	53 (311)	8 (52)	0.891 (0.400-1.985)	0.7778	61 (314)	1 (6)	0.880 (0.103-7.516)	0.9073	22 (103)	39 (235)	0.777 (0.438-1.376)	0.3865
strong positive	136 (311)	32 (52)	1.327 (0.810-2.173)	0.2607	179 (314)	10 (6)	3.177 (1.122-8.997)	0.0296	87 (103)	106 (235)	0.531 (0.366-0.770)	0.0008
Mutant p53 expression												
Yes	89 (311)	21 (52)	1.362 (0.776-2.393)	0.2821	112 (314)	3 (6)	1.507 (0.368-6.175)	0.5689	49 (103)	68 (235)	0.608 (0.393-0.942)	0.0258
No	100 (311)	19 (52)	1.068 (0.598-1.907)	0.8239	127 (314)	8 (6)	3.699 (1.243-11.007)	0.0187	60 (103)	77 (235)	0.560 (0.370-0.547)	0.0061
WT1 expression												
negative/mild positive	26 (311)	10 (52)	2.225 (1.009-4.909)	0.0476	31 (314)	1 (6)	1.867 (0.215-16.201)	0.5712	18 (103)	17 (235)	0.405 (0.200-0.819)	0.0119
strong positive	66 (311)	10 (52)	0.869 (0.418-1.806)	0.7062	75 (314)	2 (6)	1.568 (0.307-8.002)	0.5888	37 (103)	47 (235)	0.555 (0.340-0.908)	0.0170
P16 expression												
negative/mild positive	27 (311)	8 (52)	1.687 (0.722-3.940)	0.2270	35 (314)	1 (6)	1.664 (0.192-14.393)	0.6434	16 (103)	18 (235)	0.479 (0.234-0.981)	0.0443
strong positive	54 (311)	11 (52)	1.182 (0.579-2.417)	0.6459	68 (314)	2 (6)	1.649 (0.324-8.402)	0.5473	27 (103)	46 (235)	0.738 (0.434-1.255)	0.2624
ki67 expression												
negative/mild positive	19 (311)	8 (52)	2.347 (0.969-5.685)	0.0589	38 (314)	2 (6)	3.103 (0.589-16.339)	0.1816	19 (103)	19 (235)	0.432 (0.219-0.854)	0.0157
strong positive	65 (311)	13 (52)	1.173 (0.602-2.282)	0.6395	77 (314)	1 (6)	0.716 (0.084-6.082)	0.7593	28 (103)	59 (235)	0.920 (0.554-1.527)	0.7470

^a^ Adjusted for age.

**Table 3:** Association between inferred haplotypes of the *METTL5, METTL16* genes and EOC risk.

Haplotypes^a^	Cases (n=406)	Controls (n=610)	Crude OR (95% CI)	P	Adjusted OR (95% CI)	P^b^
	No.%	No.%				
GAG	132 (32.51)	201 (32.95)	1.000		1.000	
AAG	76 (18.72)	92 (15.08)	1.059 (0.738-1.521)	0.754	1.034 (0.718-1.489)	0.859
AGA	3 (0.74)	6 (0.98)	0.641 (0.158-2.600)	0.534	0.696 (0.170-2.845)	0.614
AGG	3 (0.74)	5 (0.82)	0.769 (0.181-3.263)	0.722	0.944 (0.222-4.018)	0.938
GGG	34 (8.37)	53 (8.69)	0.823 (0.512-1.321)	0.419	0.833 (0.517-1.343)	0.454
AAA	70 (17.24)	136 (22.30)	0.660 (0.466-0.936)	0.020	0.662 (0.466-0.941)	0.021
GAA	71 (17.49)	105 (17.21)	0.867 (0.605-1.243)	0.437	0.865 (0.602-1.243)	0.433
GGA	17 (4.19)	12 (1.97)	1.817 (0.846-3.903)	0.126	1.942 (0.899-4.195)	0.091

^a^ The haplotypes order was rs3769767, rs1056321, rs10190853, rs3769768, and rs11869256. ^b^ Obtained in logistic regression models with adjustment for ag

**Table 4:** Best multifactor dimensionality reduction interaction model

*Locus number*	Number of the risk factors	Test Accuracy	CVC	OR	95% CI	P
1	METTL5_rs3769767, METTL16_rs1056321, METTL5_rs10190853	0.6191	10/10	2.505	0.8889-7.0587	0.0793

^a^ The model with the maximum testing accuracy and maximum CVC was considered the best.
