# Supplementary material for: Gene polymorphisms of METTL5 and METTL16 are related to epithelial ovarian cancer risk in South China: A three-center case-control study
Source: J Cancer. 2024 Feb 4;15(6):1762–9. doi: 10.7150/jca.90379 (PMC10869976; doi:10.7150/jca.90379)
Supplement: Supplementary file 1 — Supplementary table. [file jcav15p1762s1.pdf]

Table S1. Clinical characteristics of EOC patients and healthy control subjects.

Varialble		case	control	*P*
		n=288	n=361	
Age (years)		N(%)	N(%)	0.5664
	≤53	155(53.82)	233(64.54)	
	＞53	133(46.18)	128(35.46)	
Pausimenia				
	post-menopause	206(71.53)	NA	
	pre-menopause	82(28.47)	NA	
Metastasis				
	Yes	98(34.03)	NA	
	No	171(59.38)	NA	
FIGO stage				
	I	71(24.65)	NA	
	II	49(17.01)	NA	
	III	93(32.29)	NA	
	IV	25(08.68)	NA	
Pathological grade				
	Low/middle	85(29.51)	NA	
	High	171(59.38)	NA	
Tumor number				
	Single	101(35.07)	NA	
	Multiple	117(40.63)	NA	
Tumor size (cm)				
	≤3 cm	192(66.67)	NA	
	>3 cm	72(25.00)	NA	
pregnant times				
	≤3 cm	126(43.75)	NA	
	>3 cm	162(56.25)	NA	
ER expression				
	negative/mild positive	38(13.19)	NA	
	strong positive	81(28.13)	NA	
PR expression				
	negative/mild positive	38(09.72)	NA	
	strong positive	47(16.32)	NA	
PAX8 expression				
	negative/mild positive	29（10.07）	NA	
	strong positive	72（25.00）	NA	
Wildtype p53				
	Positive	66(22.92)	NA	
	Negative	222(77.08)	NA	
Mutant p53				
	Positive	133(46.18)	NA	
	Negative	155(53.82)	NA	
WT1 expression				
	negative/mild positive	37（12.85）	NA	

strong positive	92（31.94）	NA
p16 expression		
negative/mild positive	38(13.19)	NA
strong positive	84(29.17)	NA
ki67 expression		
negative/mild positive	48(16.67)	NA
strong positive	96(33.33)	NA
